# Study on the effect of geometrical and operational parameters on performance dynamics of modified rotary blades using DEM

**DOI:** 10.1038/s41598-024-69803-8

**Published:** 2024-08-20

**Authors:** Rohit Dilip Nalawade, Krishna Pratap Singh, A. K. Roul, K. N. Agrawal, Shital Sonawane, Aman Mahore, Abhishek Patel, Mohit Kumar, Pramod Shelake, Ali Salem, Ahmed Elbeltagi

**Affiliations:** 1 ICAR-Central Institute of Agricultural Engineering, Berasia Road, Bhopal, 462038 India; 2 Indian Council of Agricultural Research (ICAR), 405-KAB-II, New Delhi, 110001 India; 3 ICAR-Central Institute of Agricultural Engineering, Berasia Road, Bhopal, 462038 India; 4 ICAR-Central Institute of Agricultural Engineering, Berasia Road, Bhopal, 462038 India; 5 Government College of Agricultural Engineering and Technology, Krishi Vidyan Sankul, Kashti, Malegaon, 423105 India; 6 ICAR-Central Institute of Agricultural Engineering, Berasia Road, Bhopal, 462038 India; 7 College of Agricultural Engineering and Post-Harvest Technology, Central Agricultural University, Imphal, Ranipool, Sikkim 737135 India; 8 Sri Karan Narendra Agriculture University, Jobner-Jaipur, India; 9 ICAR-National Research Centre for Banana, Thogamalai Road, Thayanur Post, Tiruchirapalli, Tamil Nadu 620102 India; 10 Civil Engineering Department, Faculty of Engineering, Minia University, Minya, Egypt; 11 Structural Diagnostics and Analysis Research Group, Faculty of Engineering and Information Technology, University of Pécs, Pécs, Hungary; 12 Agricultural Engineering Department, Faculty of Agriculture, Mansoura University, Mansoura, 35516 Egypt

**Keywords:** Sweepback angle, Overall mixing index, Subdomain mixing index, Disturbance intensity, Discrete element methods, Energy science and technology, Geophysics, Mechanical engineering

## Abstract

The geometric features and operational parameters of rotary blades on rotary tillers significantly impact their performance characteristics. The sweepback angle is a geometric feature of the 'L'-shaped rotary blade that has remained unexplored in previous studies. This study aimed to analyze the effect of geometrical and operational parameters on the performance dynamics of the 'L'-shaped rotary blade. The investigation was conducted using the discrete element method (DEM) and further validated through experiments conducted in a soil bin. The simulation experiment was conducted by dividing the particle bed into horizontal particle zones. The effect of the geometrical (sweepback angle) and operational parameters (forward speed, rotational speed, and depth) on the power requirement, disturbance intensity, and mixing index was studied. The novel method was adopted to determine the mixing capability of rotary blades in terms of sub-domain mixing index (SMI) and overall mixing index (OMI). The results revealed that the power requirements for a sweepback angle of 18° were 26.39% and 16.50% lower than those for sweepback angles of 6° and 12°, respectively. The sweepback angle tends to have the least effect on the overall mixing index compared to operational parameters. The average particle velocity decreased by 22.19% and 29.60% with sweepback angles of 12° and 18°, respectively, compared to the sweepback angle of 6°. The relative error during the experiment varied between 1.29% and 13.51%. It was concluded that the sweepback angle was found to be a feasible option for reducing the power requirement with good mixing indices.

## Introduction

The primary focus of developing rotary blades has been to achieve optimal performance, with a particular emphasis on reducing power requirements. The geometric shape of the rotary blade is one of the important factors that can impact the power requirement of the rotary tiller. Researchers have attempted to modify the geometric features of existing rotary blades. Saimbhi et al.^1^ focused on modeling the back surface of a C-shaped rotary blade using the Bezier surface generation approach. They modified the curvature and orientation angle of the blade at certain spots, contributing to soil compaction issues. The changes in design effectively resolved the issue of interference and improved the overall performance of the blade during tillage operations. Asl and Singh^2^ developed three kinds of rotary blades (namely, C, L, and RC-type) to minimize the energy requirement for tillage through the optimization of parameters influencing the cutting force of these blades. Matin et al.^3^ examined the impact of three different blade geometries: conventional, half-width, and straight. Their findings indicated that a straight blade required the lowest levels of torque and power. Yang et al.^4^ developed rotary tillage blades inspired by the geometric attributes of the five foreclaws of mole rats, aiming to minimize torque and energy requirements. They discovered that bioinspired blades required less torque than conventional blades. Wang et al.^5^ conducted a study aimed at improving the structural efficiency of rotary tillage blades by incorporating the badger claw toe as a biomimetic model. The study results revealed that there is a decrease in strain and stress, as well as an increase in specific strength and stiffness, in the bionic design. Zhang et al.^6^ utilized serrated structures inspired by convergent evolution to develop the front, side, and transition cutting edges of a blade. The bioinspired serrated structure resulted in a 22.25% reduction in the resistance torque.

Modeling soil-tool interactions is a complex task due to the involvement of numerous variables impacting soil behavior. The modeling process requires a comprehensive understanding of the underlying mechanisms involved. The discrete element method (DEM) has the potential to accurately capture the behavior of soil with a fair degree of accuracy. In recent years, researchers have proposed various discrete element models to better understand the phenomenon of soil-tillage tool interactions^7^. Hirasawa et al.^8^ developed a DEM model to predict the power required and the phenomenon of soil throwing in rotary tilling. The model could predict the soil throw area with an accuracy of 92–104% and the required torque with an accuracy of 83–110%. Humini et al.^9^ conducted a DEM simulation to analyze the interaction between a rotary 'C'-shaped blade and soil with straw. They concluded that the DEM provides a better understanding of the torque and forces involved in tillage. Ucgul et al.^10^ investigated the mixing capability of a rotary spader in the upper surface layer through image processing and discrete element modeling. They reported that field experiments were unable to analyze forward soil displacement, but this was successfully simulated and analyzed with a DEM. Zhao et al.^11^ conducted a simulation study to examine the impact of different edge curve geometries of rotary tiller blades on the torque requirement and resultant soil disturbance through DEM. Zhao et al.^12^ investigated a counter-rotating excavation device used for harvesting Cyperus esculentus by conducting discrete element simulation tests on Cyperus esculentus agglomerates under distinct soil layers. The optimized structural parameters of the excavation device reduced the working resistance and torque significantly. Zhu et al.^13^, on the other hand, studied the throwing performance of bionic rotary blades during Cyperus esculentus harvesting. The bionic rotary blade with the excavation edge and surface design had the best operational performance. Zhai et al.^14^ studied the operational and structural parameters of the vertical axis rotary tiller using DEM simulation to improve its operational quality. They reported that the soil crushing effect can be improved by increasing the contact area between the tool and the soil by increasing the bending angle of the tool. Zhang et al.^15^ studied rotary curved blade tillage cutting simulations to predict energy consumption for different combinations of operational parameters. They reported a good agreement between the simulation and the measured results. Patidar et al.^16^ investigated the effect of operational conditions on the torque and draft requirement of the vertical axis rotary tiller using DEM simulations. They found that increasing the ratio of peripheral velocity to the blade tip speed resulted in a significant reduction in the negative draft and average torque requirement. There are also other recent studies which focus on advanced techniques in this regard such as Zhang and Ma^17–19^.

The abovementioned studies have highlighted that geometrical features and operational parameters influence the performance of rotary tiller blades. Most of these studies are associated with curved and ‘C’ rotary blades. Despite widespread use and relatively high power requirements, only a few researchers have explored the geometric features of 'L'-shaped rotary blades. The sweepback angle is a geometric feature of the 'L'-shaped rotary blade that previous studies have often overlooked. Certain rotary blade manufacturers claim that a sweepback angle of a few degrees can reduce the load on the tractor and increase fuel efficiency. However, there is no scientific evidence available to support their claim. Therefore, this study aimed to investigate the influence of sweepback angle and operational parameters on the performance of the 'L'-shaped rotary blade using the discrete element method (DEM) and validate it through soil bin experiments.

## Materials and methods

This section deals with the development of L-shaped blades, the DEM-based soil model, the simulation of developed blades to analyze their performance under different experimental conditions, and its validation through soil bin experiments.

### Development of ‘L’ shaped rotary blade

**Figure 1:**
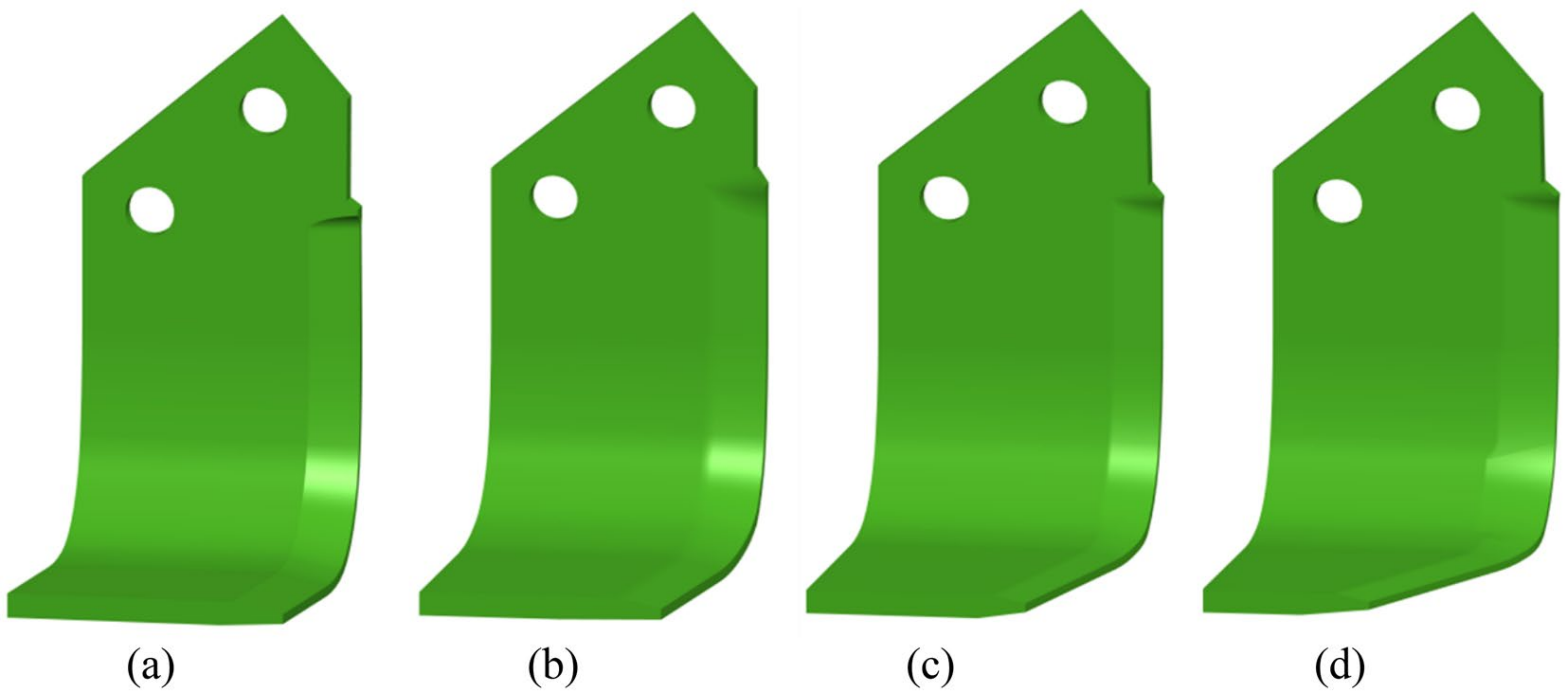
CAD geometries obtained by 3D blue light scanning (**a**) 0°, (**b**) 6°, (**c**) 12°, and (**d**) 18°.

The primary objective was to develop a conventional L-shaped blade that would decrease penetration resistance by adjusting one of its key geometric features, the sweepback angle. This angle refers to the angle formed by the cutting edge of the rotary blade with the soil surface at the beginning of penetration. The ‘L’-shaped rotary blade was developed based on the work presented by He et al.^20^. They reported that the attack surface area significantly influences the vertical penetration of the blade into the soil. The attack surface area refers to the area of the blade that comes into contact with the soil particles as it enters into the soil mass. A decrease in the attack surface area results in a reduced vertical force (or penetration resistance). It was assumed that the attack surface area decreases as the sweepback angle increases. Based on these assumptions, blades with sweepback angles of 6, 12, and 18° were fabricated. To ensure the accuracy of the dimensions of the shape of the modified blades, a 3D blue light scanner (Make: GOM and Model: Atos Core 300) was used to obtain the CAD geometries of the developed blades, as shown in Fig. 1. The gross surface areas of the developed blades were 45,726, 44,354, 43,561, and 41,928 mm^2^ for sweepback angles of 0, 6, 12, and 18°, respectively.

### Development of the DEM-based soil model

The soil was modeled using the hysteretic spring contact model^21^ and the linear cohesion V2 model^22^. The combination of the hysteretic spring contact model with a linear cohesion model effectively captures two key properties of agricultural soil, i.e., plasticity and cohesion. Additionally, these models require fewer input parameters compared to other elastoplastic models, making them potentially the most appropriate contact models for simulating agricultural soils^23^.

The hysteretic spring contact and linear cohesion model V2 parameters require 13 major input parameters. It was very difficult to calibrate the values of all of these parameters. According to Nalawade et al.^23^, only the coefficient of friction soil‒soil, coefficient of friction soil-metal, yield strength (soil)) and energy density have a significant effect on the model output. Therefore, calibration of only significant model parameters was performed using a cone penetration test, and parameter values were optimized using a central composite design^24^. The parameter values calibrated and selected from previous literature are listed in Table 1. These parameters are used for the simulation experiments involved in this study.

**Table 1:** Calibrated DEM parameters.

Sr. No	Parameters	Unit	Value	Reference
1	Solid density	${\text{kgm}}^{-3}$	2600	^25^
2	Shear modulus (soil)	Pa	1 × 10^7^	^23^
3	Shear modulus (steel)	Pa	7.9 × 10^10^	^26^
4	Coefficient of restitution soil‒soil	–	0.3	^27^
5	Coefficient of friction soil‒soil	–	0.42	Calibrated
6	Coefficient of rolling friction soil‒soil	–	0.4	^28^
7	Coefficient of restitution soil-steel	–	0.05	^29^
8	Coefficient of friction soil-steel	–	0.35	Calibrated
9	Coefficient of rolling friction soil-steel	–	0.2	^30^
10	Energy density	J.m^-3^	28	Calibrated
11	Stiffness factor	–	0.7	^23^
12	Damping coefficient	–	0.05	^31^
13	Yield strength	MPa	1.12 × 10^6^	Calibrated
14	Time step	s	10% of Rayleigh Time Step	^32^

### Single flange rotary tiller experiment using DEM

**Figure 2:**
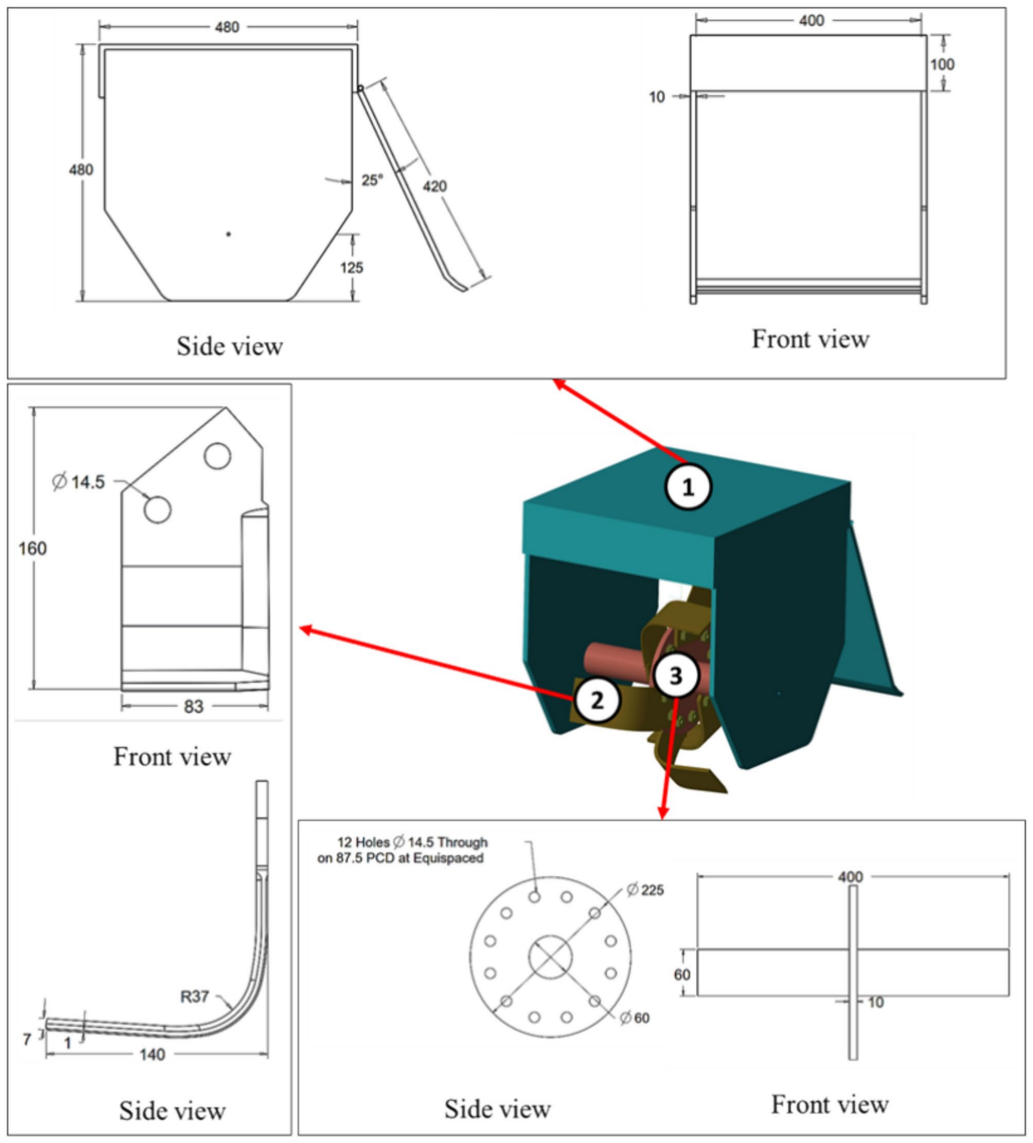
Specifications of the single flange rotary tiller model: (1) outer cover with an adjustable back cover, (2) blade mounting flange with shaft, and (3) ‘L’-shaped blade.

The developed blades were tested in a controlled soil bin environment to easily control various experimental parameters that can be difficult to measure during field tests. However, due to power and space constraints in the soil bin, it was not possible to study the rotary tiller at its full width. As a result, a single-flange rotary tiller model, as illustrated in Fig. 2, was fabricated to perform soil bin tests with the developed blades.

According to Zeng et al.^33^, the DEM makes it possible to study dynamic aspects of soil tool interactions more easily compared to physical experimentation. Therefore, before directly committing the modified blades to soil bin experiments, it was conceivable to study their dynamic aspects using the calibrated DEM model and then validate the results using soil bin experiments.

The simulation experiment for the single flange rotary tiller was carried out using EDEM 2021 software on HP computer with an Intel Core i9-9900 K CPU @3.6 GHz and 32 GB of RAM. EDEM software is used to model the behavior of bulk materials. It consists of three modules: Creator, Simulator, and Analyst. The Creator used to build the bulk material model, import CAD geometries, and assigns the geometry motions. The Simulator is used to set up the simulation time, timestep, and other parameters. The analyst was used to analyze the simulation outputs such as particle forces, velocities, and accelerations, as well as the torque and forces acting on the geometries.

**Figure 3:**
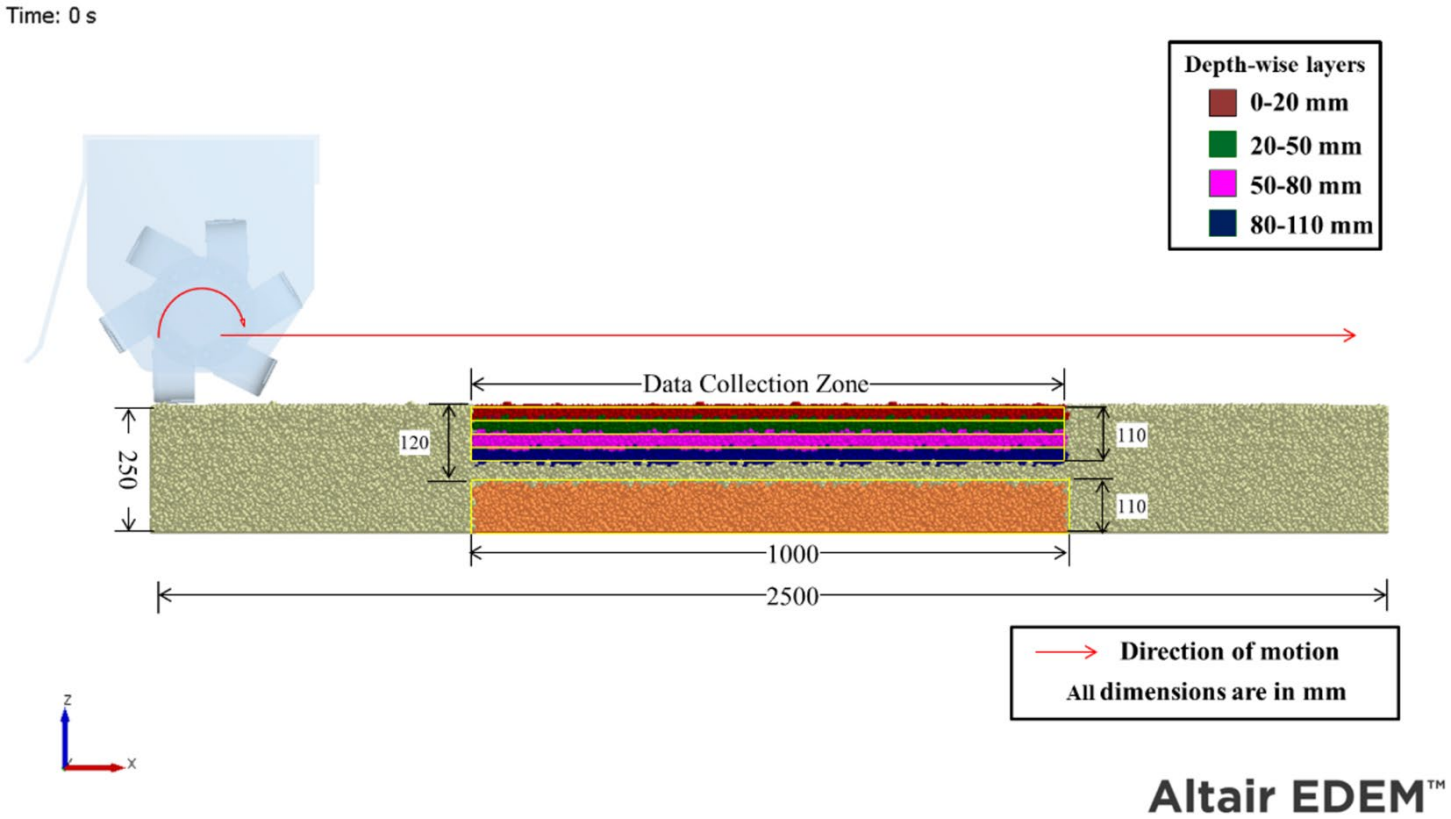
Simulation settings of the single flange rotary tiller model.

A virtual soil bin model, measuring 2500 × 1500 × 250 mm, was created for the simulation (Fig. 3). A length of 2500 mm ensured that the operation of the rotary tiller stabilized enough to obtain meaningful output data. The simulations were conducted using the calibrated set of model parameters (Table 1). The particle bed was created by randomly generated multispherical particles with an average diameter of 5 mm and a range spanning from 4 to 6 mm. The block factory feature in the EDEM software was employed to generate a virtual soil bin. Before commencing the actual simulation, the particles were permitted to settle until their velocity and kinetic energy dropped below 0.05 m.s^−1^ and 0.01 J, respectively.

The simulation settings for the simulation of the single flange rotary tiller model are shown in Fig. 3. The CAD geometry of the rotary tiller blade assembly and outer cover (Fig. 2) was imported into the software. The imported geometry was positioned at one end of the soil bin. The central section, measuring 1000 mm in length and 800 mm in width, was selected as the data collection zone. This choice was made because it appeared that the simulation had stabilized approximately 750 mm from the beginning of the particle bed. The data collection zone was horizontally divided into four distinct layers. The topmost layer measured 20 mm, while all other subsequent layers measured 30 mm each. The particles within these sections were selected using a manual particle selection tool and colored accordingly to differentiate between particles of different layers.

To provide the desired depth to the single flange rotary tiller model, both rotary tiller flange assembly and cover were initially given a downward linear velocity kinematic for the period of 0.1 s and linear rotational kinematic to the blade assembly for the period equal to the total simulation time. As soon as the model (blade assembly and cover) attained its desired depth (after 0.1 s), it was given a forward linear velocity kinematic for the period equal to the total simulation time. In this way, both translational and rotational velocities were given to the blade assembly, while the cover was only provided with translation velocity.

The DEM study of a single flange rotary tiller for developed blades was planned using a full factorial, completely randomized design (FFCRD) using Design Expert software. The layout for 80 experimental runs, along with three replications of each (80 × 3 = 240 runs), was prepared based on the FFCRD. The one-way ANOVA analysis technique was used to analyze the data and independently compare the means across different levels of each parameter. Multiple pairwise comparison tests were also performed to check the significance of the levels of the independent parameters. The experimental parameters and their respective levels are presented in Table 2. The experiment was conducted with the different sweepback angles, forward speeds, rotational speeds, and depths, and their effect on power requirement, disturbance intensity, and mixing index was studied.

**Table 2:** Parameters and their levels for the single flange rotary tiller experiment.

Sr. No	Parameters	Unit	Levels	
			Low (− 1)	High (+ 1)
1	Sweepback angle	Degree	6	18
2	Forward speed	km h^−1^	0.30	1.20
3	Rotational speed	rpm	200	300
4	Depth	mm	20	80

The forward speed range used in the laboratory experiment was less than that used in the field experiments. This was because there was limited power available to operate the single flange model with a forward speed range compared to that used for full-width rotary tillers in the field. Therefore, the power constraint required the use of a lesser forward speed range in the laboratory experiment. Celik and Altikat^34^ also experimented with a similar range of forward speeds.

A detailed description of the methodology adopted for the determination of each dependent variable mentioned above is presented in the following subsections.

#### Power requirement

The average power required by the single-flange rotary tiller model is a function of the rotational speed and torque acting on the blade mounting shaft. The average torque required by the blade assembly was determined by assuming that the line perpendicular to the direction of travel and passing through the center of gravity of the blade assembly is the axis of rotation of the flange. The torque values acquired before and after the data collection zone were ignored, and the torque values obtained from the data collection zone were used for further calculations. The power required by the single rotary tiller model under the different experimental conditions was calculated with the following equation:

1 $${\text{P}} = \frac{{2\pi {\rm{N}}_{\text{r}} {\rm{T}}_{\text{r}} }}{{60}},$$

where P is the power required by the single flange rotary tiller model (W); ${N}_{r}$ is the blade rotational speed (rpm); and ${T}_{r}$ is the torque acting on the rotary tiller shaft (N.m.).

The uncertainty analysis of the measured power requirement was performed using the methodology suggested by Rahmatian et al.^35^ and Rahmatian et al.^36^.

The power requirement of the rotary tiller is the function of rotational speed and torque acting on the rotary tiller shaft:

2 $$\text{P}\hspace{0.17em}=\hspace{0.17em}f\left[{\text{N}}_{\text{r}} ,{\text{T}}_{\text{r}}\right].$$

The uncertainty of the rotary tiller power requirement is obtained by partial differentiation, mentioned in the following equation

3 $$\Delta P= \sqrt{{\left(\frac{\partial f}{\partial {\text{N}}_{\text{r}}}\Delta {\text{N}}_{\text{r}}\right)}^{2}+{\left(\frac{\partial f}{\partial {\text{T}}_{\text{r}}}\Delta {\text{T}}_{\text{r}}\right)}^{2}}$$

The uncertainty in the measurement of the power requirement of the rotary tiller is determined by Eq. (4), which is obtained by dividing Eq. (3) by (2).

4 $$\frac{\Delta P}{P}=\boldsymbol{ }\sqrt{{\left(\frac{\Delta {\text{N}}_{\text{r}}}{N}\right)}^{2}+{\left(\frac{\Delta {\text{V}}_{\text{r}}}{V}\right)}^{2}}$$

The speed control accuracy of the variable frequency drive (VFD) was 3%, while the torque measurement accuracy of the torque transducer was 0.5%. The total uncertainty was calculated as 3.04%.

#### Disturbance intensity

The disturbance intensity of the soil particles is a function of the disturbance ability of rotary tiller blades^26^. The disturbance intensity of soil can be quantified by determining the average velocity attained by soil particles when the high-speed rotary tiller blades interact with the soil, causing bonds between them to fail due to impact and shear, lift upward, and eventually strike on the outer cover of the rotary tiller. The average velocity of the particles induced by their interaction with the blades was recorded within the data collection zone.

#### Mixing index

The ability to intermix soil particles from different layers is an important characteristic of rotary tillers. To determine the mixing index, the depth of the soil was divided into four different layers (0–20, 20–50, 50–80, and 80–110 mm), as shown in Fig. 3. The positional coordinates of one thousand selected particles from each layer were recorded before and after the rotary tiller operation. These positional coordinates were then used to determine the movement of particles concerning their initial position. The methodology suggested by Cho et al.^37^ was used to calculate the mixing index based on the positional data of soil particles. This approach involves obtaining mixing information from individual subdomains (in this case, different layers) and combining them to determine the overall mixing index. The subdomain mixing index can be calculated by taking the arithmetic mean of the individual fractions of each type k particle, except for the fraction of the majority type, within each subdomain. The following equation gives the subdomain mixing index (SMI):

5 $$\text{SMI}= \frac{1}{\rm{Q}-1}\left(\sum_{\text{k}=1}^{\rm{Q}}{\text{P}}_{\rm{ki}}-1\right),$$

where SMI is the subdomain mixing index, Q is the number of types of particles, k is the type of particle, and ${P}_{ki}$ is the fraction of particle type k.

The total or overall mixing index was calculated based on the subdomain mixing index by averaging each mixing index of all subdomains. The mathematical relation used to calculate the overall mixing index (OMI) is given by the following equation:

6 $$\text{OMI}= \frac{1}{{\text{N}}_{\text{i}}}\sum_{\text{i}=1}^{\text{M}}\left[\text{SMI}\sum_{\text{n}=1}^{\text{Q}}{(\text{n}}_{\text{ki}})\right],$$

where OMI is the overall mixing index, ${N}_{i}$ is the number of particles in a subdomain, and M is the number of subdomains.

### Validation of the DEM single flange rotary tiller experiment through soil bin experiments

The developed single flange rotary tiller DEM model was validated through physical soil bin experiments. The detailed procedure adopted for the validation experiment is described in the following subsections.

A soil bin experiment was conducted to validate the simulation experiment of the designed rotary tiller blades. As previously stated, it was not possible to conduct experiments using a full-width rotating tiller unit due to space and power availability constraints. Therefore, to perform all validation studies, a single flange rotary tiller model (Fig. 2), with provisions for mounting six blades (three blades on one side and three on the other), was fabricated. The torque experienced by the blade shaft was measured by using a torque transducer (Make: HBM Model: T22, Capacity: 1 kN.m). It was installed between the output shaft of the motor and the driving shaft.

A rectangular instrumented soil bin with dimensions of 17 × 3 × 1 m was used to perform all the experiments. It consists of Vertisols (black cotton soil) with 61.27, 27.15, and 11.59% clay, silt, and sand, respectively. The average moisture content of the soil during the experiment was 14 ± 2% (wb). The cone index (CI) of the soil bed was kept constant at 850 ± 50 kPa for all experimental runs.

**Figure 4:**
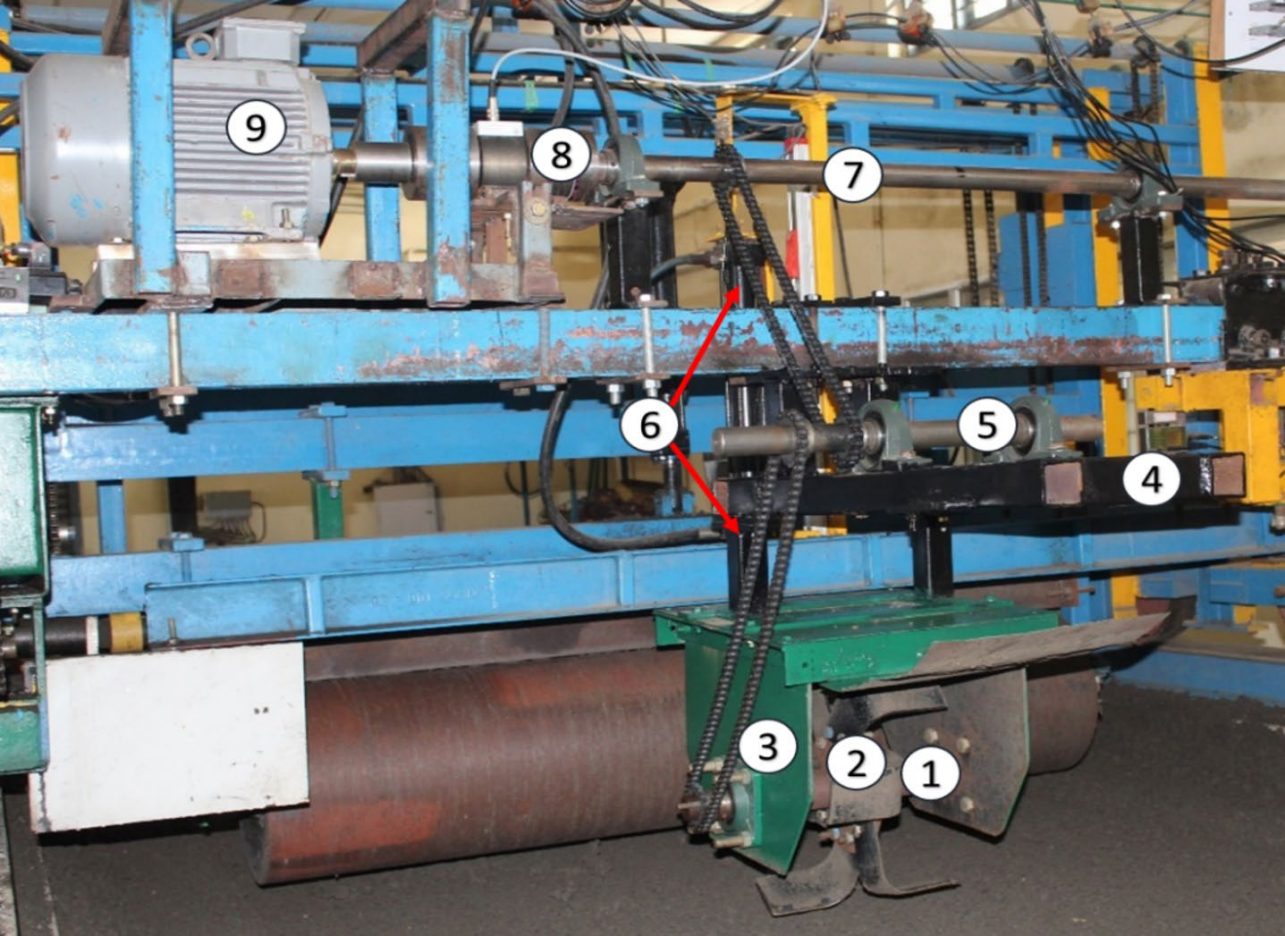
Soil bin experiment setup (1) hallow shaft with blade flange (2) blades (3) outer cover (4) idle shaft frame (5) idle shaft (6) chain drives (7) driving shaft (8) torque transducer (9) electric motor.

The soil bin experiment setup is depicted in Fig. 4. The soil bin test was performed by referring to the procedure outlined by Nalawade et al.^23^. To prepare the soil bed according to the test conditions, the soil was first sprayed with the required amount of water using a water dispensing system. This allowed the soil to reach a moisture level within a specified range. A front-mounted rotary tiller was then used to mix the soil properly. The soil was subsequently compressed using a compaction roller to achieve an average cone index of 850 ± 50 kPa. A hydraulically powered cone penetrometer installed on the frame of a soil processing trolley was used to measure the cone index.

To mark the zero-depth position, the rotary tiller model was lowered using a depth adjustment wheel until the lower surface of the blade just touched the soil bed surface. The depth adjustment wheel was then locked to maintain the zero-depth setting. The required depth was provided to the rotary tiller by lowering it at the depth adjustment gap provided at one end of the soil bin.

It was not possible to measure and validate all the dependent parameters studied in the DEM simulation experiment. Therefore, only the average torque experienced by the rotary tiller shaft was considered for the validation. To observe the effect of these parameters on the torque requirement, the experiment was planned using a full factorial completely randomized design (FFCRD) with three replications of each experimental run.

To validate the DEM experiment, the relation between the simulated and observed torques was evaluated using the relative error. The relative error was computed using the following formula:

7 $${\text{RE}}_{torque}= \left|\frac{{T}_{\text{o}}-{T}_{\text{s}}}{{T}_{\text{O}}}\right|\times 100,$$

where ${\text{RE}}_{torque}$ is the relative error between the observed and simulated torques (%), ${T}_{\text{o}}$ is the observed torque value (N.m.), and ${T}_{\text{s}}$ is the simulated torque value (N.m.).

## Results

The developed blades were assessed using single flange rotary tiller model DEM simulation. The dependent parameters, i.e.*,* power requirement, disturbance intensity (average particle velocity) and mixing index, were analyzed to study the performance of the developed blades. The ANOVA results for the effect of the independent parameters on the dependent parameters for the single flange rotary tiller experiment are shown in Table 3.

**Table 3:** ANOVA showing the effect of independent parameters on dependent parameters for the single flange rotary tiller experiment.

Source	Power requirement, W	Average particle velocity, m.s^-1^	Mixing index
	‘F’ values with significance		
Model	549.63***	232.89***	801.47***
A	610.67***	254.78***	96.75***
B	1228.24***	2587.14***	246.02***
C	64.69***	22.19***	158.41***
D	3396.24***	320.24***	10,341.92***
A × B	6.15*	37.90***	4.66*
A × C	10.52**	3.25^NS^	0.0463^NS^
A × D	16.18***	2.54^NS^	0.0564^NS^
B × C	21.33***	7.83**	0.0523^NS^
B × D	65.94***	0.0192^NS^	2.63^NS^
C × D	76.29***	5.08*	0.3292^NS^
A^2^	–	18.87***	54.02***
B^2^	–	0.5168^NS^	0.0344*
C^2^	–	0.0082^NS^	0.7192^NS^
D^2^	–	0.0144^NS^	314.95***
Lack of fit	1.38^NS^	1.32^NS^	1.37^NS^
Mean	1262.97	0.0215	0.47
R^2^	0.96	0.93	0.98

*NS* Non-significant.

A = Sweepback Angle, B = Forward Speed, C = Rotational Speed, D = Depth.

*Significant at 5% level of significance.

**Significant at 1% level of significance.

***Significant at < 0.1% level of significance.

### Effect of independent parameters on the power requirement

It can be seen from the ANOVA results in Table 3 that the model for the power requirement was highly significant (P < 0.001). The coefficient of determination (R^2^) value of 0.96 confirms good agreement between the independent and dependent parameters. The lack of fit for the model was significant (P = 0.0505). Thus, the model was adequate to explain the effect of independent parameters on dependent parameters. The sweepback angle, forward speed, rotational speed, and depth had a highly significant effect (P < 0.001) on the power requirement. The 'F' values indicate that depth had the most significant impact on the power requirement, while rotational speed had the least impact. The order of influence of the independent parameters on the power requirement is as: depth > forward speed > sweepback angle > rotational speed.

**Figure 5:**
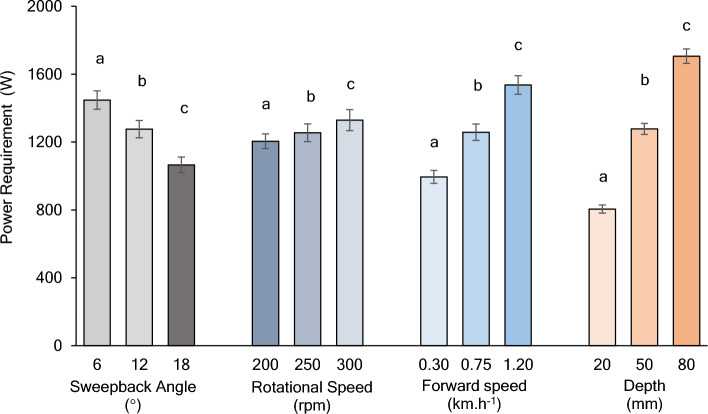
Effect of rotational speed, sweepback angle, forward speed, and depth on the power requirement of a single flange rotary tiller.

Figure 5 illustrates the impact of various operating parameters on the power requirement of the single-flange rotary tiller model. It was evident from the results that the power requirement was highest for a sweepback angle of 6°, whereas it was lowest for a sweepback angle of 18° across the range of tested operating conditions. The power requirements for a sweepback angle of 18° were 26.39% and 16.50% lower than those for sweepback angles of 6° and 12°, respectively. The post hoc Tukey’s (b) test revealed that the power requirement was significantly different for all levels of the independent parameters.

The rotational speed of the rotary tiller has a positive correlation with the power requirement, which increases with increasing rotational speed. The maximum power requirement was observed for the rotational speed of 300 rpm, while the minimum power requirement was recorded at 200 rpm across the range of operating conditions. The power requirement increased by 10.32% when the rotational speed increased from 200 to 300 rpm.

The power requirement increased with an increase in forward speed. The maximum power requirement was observed with a forward speed of 1.20 km.h^−1^, while the minimum was recorded at 0.30 km.h^−1^ across the range of operating conditions. The power requirement increased by 54.48% when the forward speed increased from 0.30 to 1.20 km.h^−1^.

The power requirement rapidly increased with increasing depth of operation. Across the range of operating conditions, the highest power requirement was observed at a depth of operation of 80 mm, while the lowest was recorded at a depth of 20 mm. The power requirement at an 80 mm depth of operation was 111.92% greater than that at a 20 mm depth of operation.

### Effect of independent parameters on disturbance intensity

The disturbance intensity of the particles due to the action of the rotary tiller was measured by analyzing the average particle velocity within the data collection zone. The ANOVA results in Table 3 show that the model for average particle velocity is highly significant (p < 0.001). A coefficient of determination (R^2^) of 0.93 indicates an excellent correlation between the independent parameters and the average particle velocity. Additionally, the model had a non-significant lack of fit (P = 0.0792). The main effects, including sweepback angle, forward speed, rotational speed, and depth, significantly impacted the average particle velocity (P < 0.001). Tukey’s (b) test revealed that the average particle velocity was significantly different for all the levels of independent parameters. The 'F' values indicated that forward speed had the greatest effect on the average particle velocity, while rotational speed had the least effect. The order of significance of independent parameters is forward speed > depth > sweepback angle > rotational speed.

**Figure 6:**
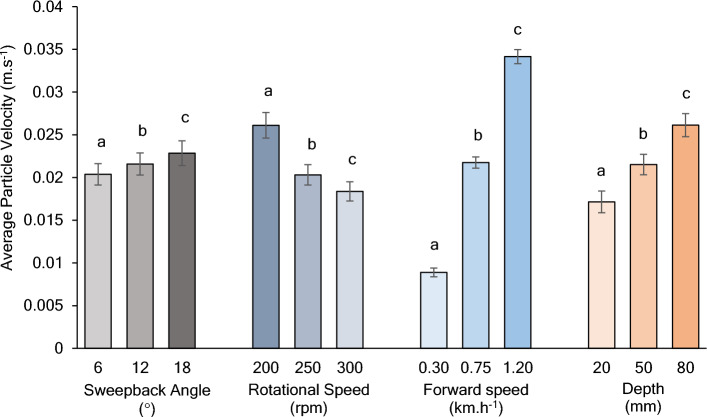
Effect of rotational speed, sweepback angle, forward speed, and depth on average particle velocity.

It is evident from Fig. 6 that the average particle velocity tends to decrease with increasing sweepback angle. The average particle velocity decreased by 22.19 and 29.60% with sweepback angles of 12 and 18°, respectively, compared to the sweepback angle of 6°.

The average particle velocity increases with increasing rotational speed of the blades. Therefore, it can be inferred that the disturbance intensity increases with increasing rotational speed. The average particle velocity at 250 and 300 rpm was observed to increase by 5.89 and 12.13%, respectively, compared to the average particle velocity at 200 rpm.

The forward speed of the rotary tiller showed a positive correlation with the average particle velocity. Thus, the particle disturbance intensity tends to increase with increasing forward speed of the rotary tiller. The average particle velocity for forward speeds of 0.75 and 1.20 km.h^−1^ tends to increase by 144.52 and 283.81%, respectively, compared with that for a forward speed of 0.30 km.h^−1^.

The depth of operation was found to have a positive correlation with the average particle velocity. As a result, the particle disturbance intensity tends to increase with increasing rotary tiller depth. The average particle velocity at depths of operation of 50 and 80 mm tends to increase by 25.44 and 52.35%, respectively, compared to a 20 mm depth of operation.

### Effect of independent parameters on the mixing index

The mixing index of rotary tiller blades with different sweepback angles was studied by horizontally dividing the data collection zone. One thousand soil particles from each vertical zone were manually selected and colored differently (according to the vertical zone) to distinguish them from one another. Particle positions before and after rotary tiller operation were recorded. Based on this information, the mixing index for each particle zone was calculated.

The ANOVA results in Table 3 revealed that the model for the mixing index (MI) was highly significant at a level of 0.1%. A coefficient of determination (R^2^) of 0.98 indicated a strong correlation between the independent parameters and the mixing index. Moreover, the lack of fit was also found non-significant (P = 0.0655). All the main effects, namely, sweepback angle, rotational speed, forward speed, and depth, had a significant effect (P < 0.001) on the mixing index. Tukey’s (b) test revealed that the mixing index was significantly different for all the levels of independent parameters. The 'F' values in the ANOVA table show that depth has the greatest effect on the mixing index, while the sweepback angle has the least effect. The order of significance of independent parameters is depth > forward speed > rotational speed > sweepback angle.

**Figure 7:**
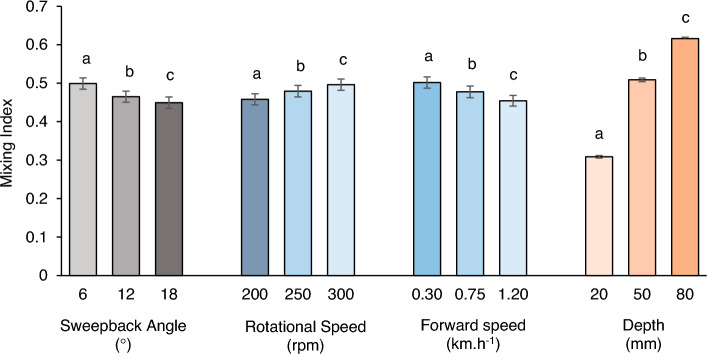
Effect of rotational speed, sweepback angle, forward speed, and depth on the mixing index.

The results revealed that there was a negative correlation between the sweepback angle and the mixing index. The mixing index tends to decrease with increasing sweepback angle (Fig. 7). Specifically, the mixing index tended to increase by 6.83 and 9.96% when the sweepback angle increased to 12° and 18°, respectively, from its lowest value of 6°.

Figure 7 shows that the mixing index had a positive correlation with the rotational speed. An increase in rotational speed increased the mixing index. Specifically, as the rotational speed increased from 200 to 250 rpm and 300 rpm, the mixing index increased by 4.63 and 8.30%, respectively.

Similarly, the mixing index was also influenced by the forward speed of the rotary tiller. As the forward speed increased, the mixing index tended to decrease. The mixing index increased by 4.82 and 9.44% when the forward speed was increased to 0.75 and 1.20 km.h^−1^, respectively, from its minimum value of 0.30 km.h^−1^.

As the depth of operation increases, the mixing index tends to increase. It was found that the mixing index increased by 64.81 and 99.54% when the depth of operation increased to 50 and 80 mm, respectively, from its minimum value of 20 mm.

**Figure 8:**
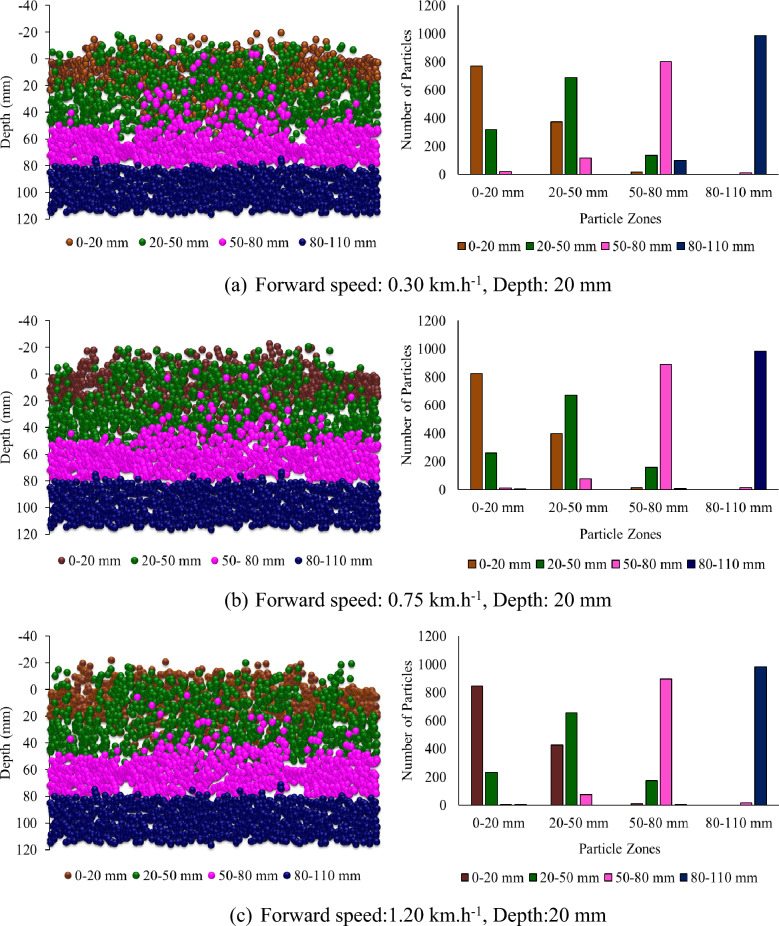

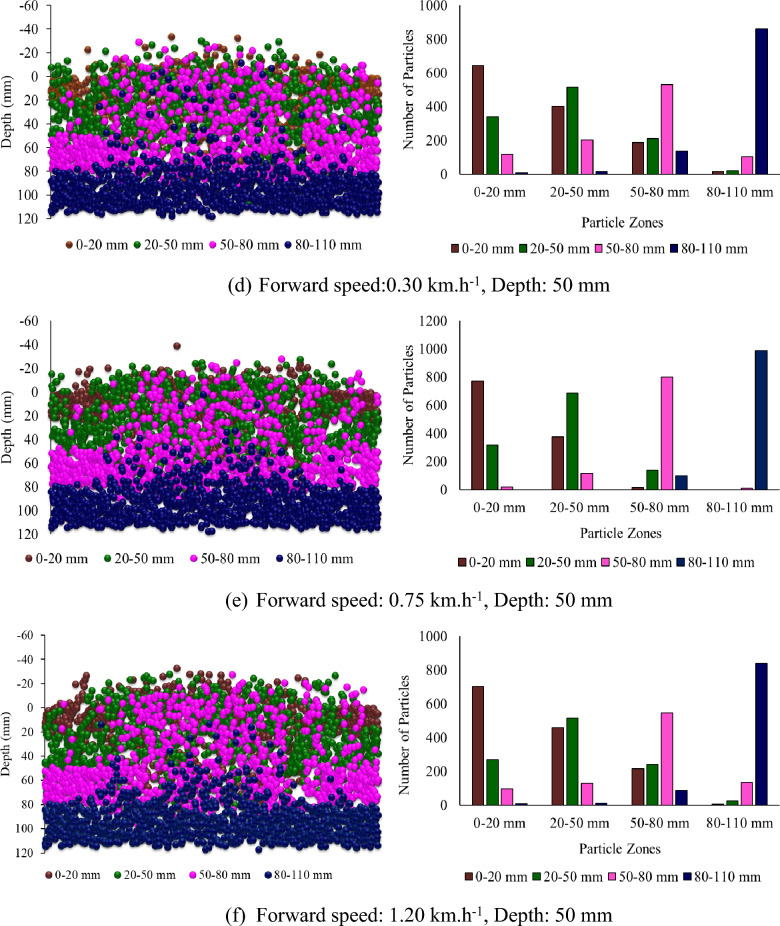

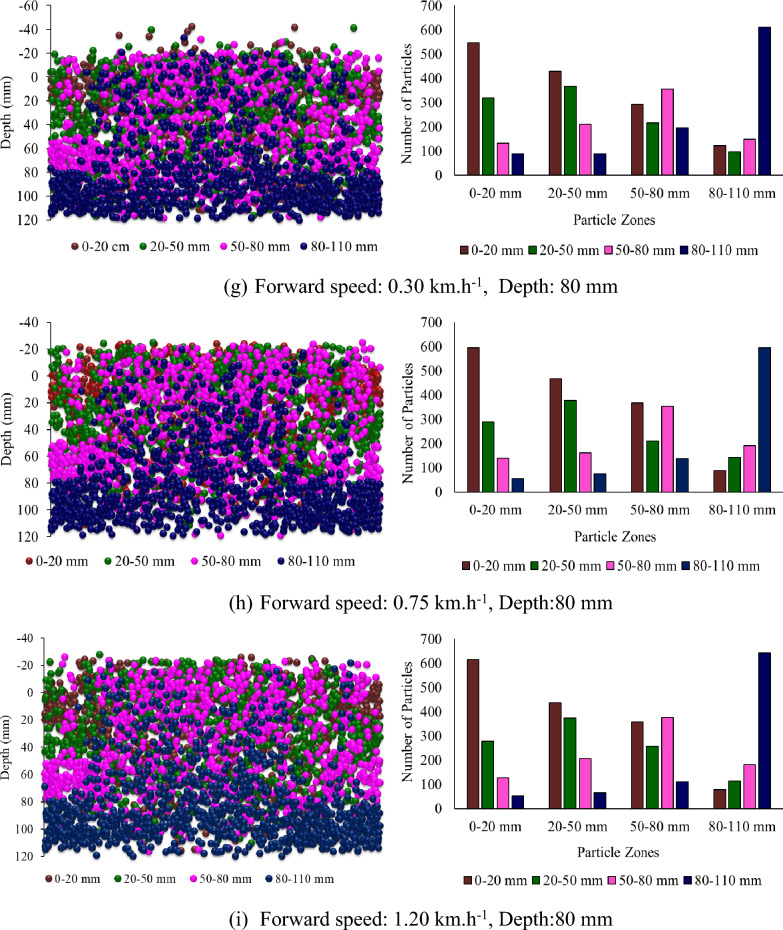
Particle Z position coordinates from different particle zones plotted against depth at different forward speeds and depths for a sweepback angle of 12° and rotational speed of 250 rpm.

Figure 8 shows the Z-positions of the different particle zones plotted against depth and the number of particles of each kind in each zone after the operation of the rotary tiller at different, forward speeds and depths at a sweepback angle of 12° and a rotational speed of 250 rpm. It was reported that at lower forward speeds, few particles from deeper particle zones moved into the upper zones. In contrast, few particles moved into deeper zones from the particle zone where they were already present (Fig. 8). However, as the forward speed increased, the interzone particle movement was not as intense as that observed when the forward speed was low. Therefore, the overall mixing index decreased with increasing forward speed.

**Figure 9:**
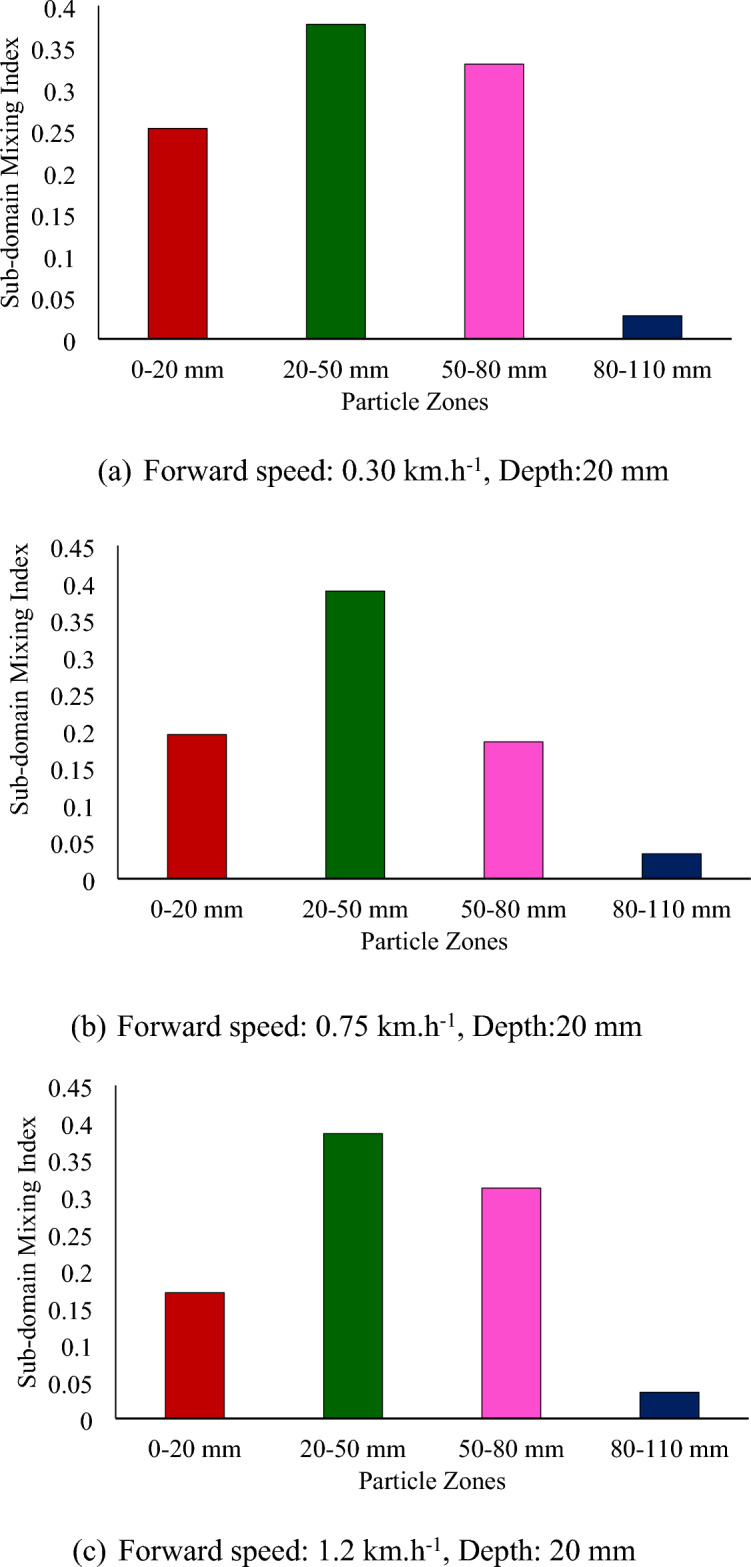

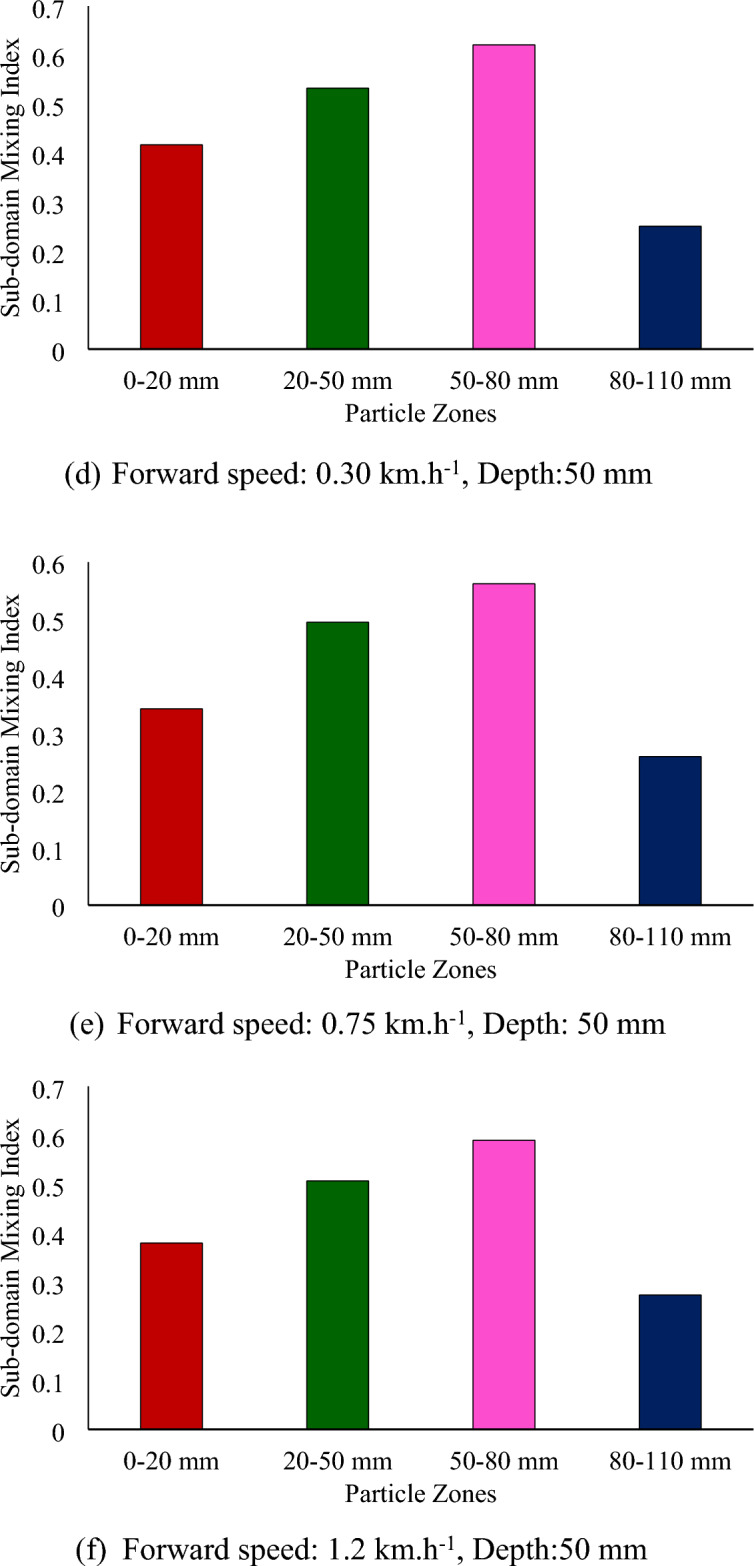

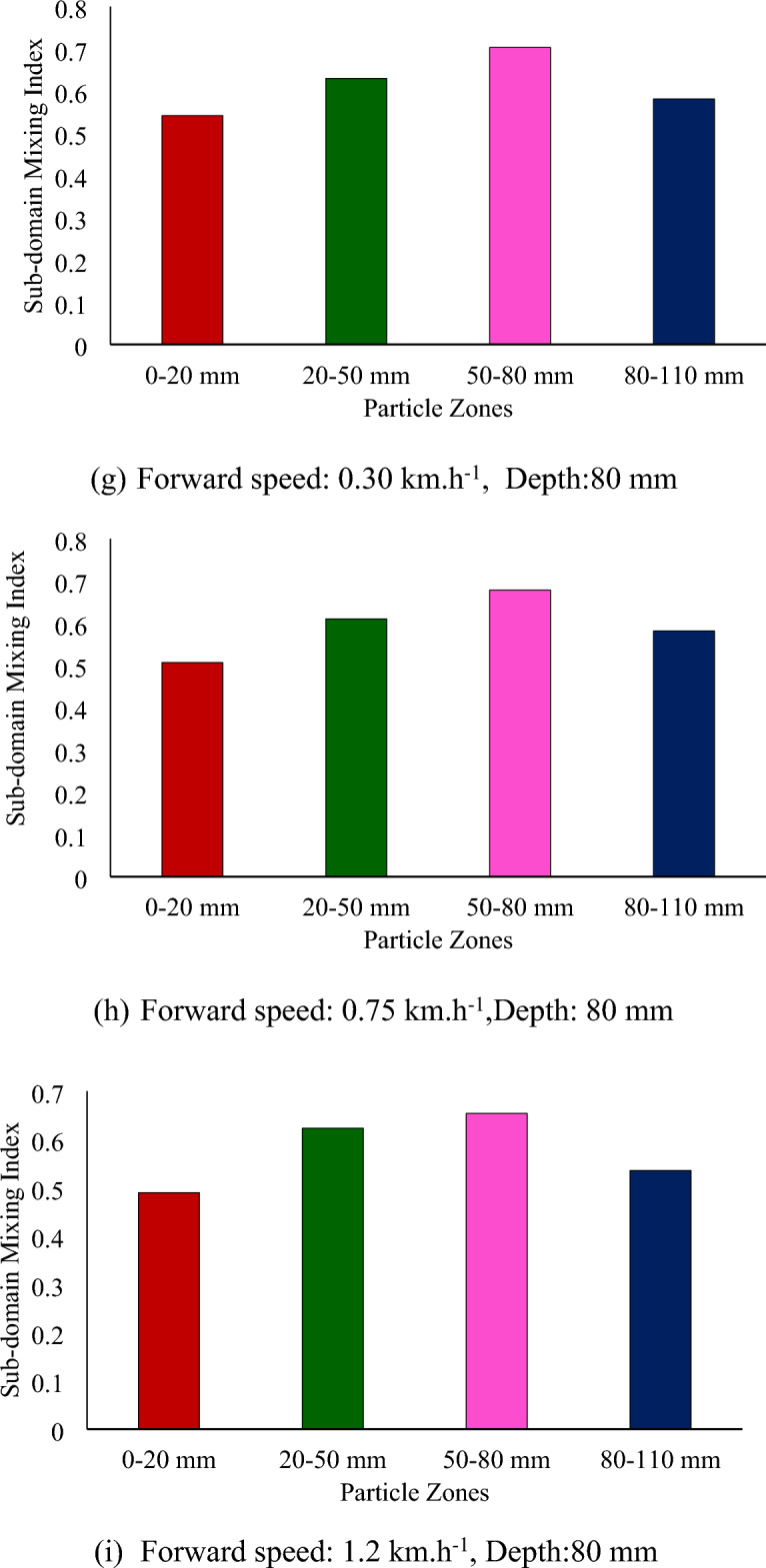
Subdomain mixing index of soil particles after operation of a rotary tiller at forward speed and depth, respectively, for sweepback angle of 12° and rotational speed of 250 rpm.

Figure 9 shows the subdomain mixing index at different forward speeds and depths for a sweepback angle of 12° and rotational speed of 250 rpm. There is a slight decrease in the subdomain mixing index of each zone with increasing forward speed (Fig. 9). As the mixing index is calculated based on the subdomain mixing index, it also tends to decrease with increasing forward speed. The depth of operation was found to be the most influential parameter affecting the mixing index. It has a positive correlation with the mixing index. As the depth of operation increases, the mixing index tends to increase (Fig. 8). The mixing index increased by 64.81 and 99.54% when the depth of operation increased to 50 and 80 mm, respectively, from its minimum value of 20 mm.

From Fig. 8a through c, it is evident that at a depth of operation of 20 mm, the top three particle zones, namely, 0–20 mm, 20–50 mm, and 50–80 mm, were disturbed, while the deeper particle zone of 80–110 mm remained undisturbed. This is further supported by Fig. 9a through Fig. 9c, which show that the subdomain mixing index of the top three zones was significantly greater than that of the 80–110 mm particle zone. It was also observed that the 20–50 mm particle zone had the highest subdomain mixing index when the rotary tiller was operated at a depth of 20 mm.

Similarly, Fig. 8d through f shows that for a depth of operation of 50 mm, the particles from the first three zones were completely disturbed, while the particles from the deepest zone (80–110 mm) were not completely disturbed. This is also evident in Fig. 9d through f, where the subdomain mixing indices of the top three zones were comparable to each other, while they were much lower for the 80–110 mm particle zone. It can also be observed from Fig. 9 that the 50–80 mm particle zone had the highest subdomain mixing index when the rotary tiller was operated at a depth of 50 mm. As shown in Fig. 9g through i, operating the rotary tiller at a depth of 80 mm resulted in the disturbance of particles from all zones, leading to a good mixing index of particles in each subdomain or particle zone. This can be observed in Fig. 9, where the subdomain mixing index of all the particle zones was found to be comparable.

The subdomain mixing index of the 80–110 mm particle zone also improved compared to that of the 50 mm particle zone. However, as observed in the case of the 50 mm depth of operation, the 50–80 mm particle zone remained the zone with the highest subdomain mixing index.

### Validation of the DEM single flange rotary tiller experiment under a soil bin

The validity of the DEM single-flange rotary tiller experiment was confirmed through soil bin experiments, which were conducted similarly to the simulation experiment. However, only the torque parameter was considered to confirm the validity of the DEM single-flange rotary tiller experiment.

**Figure 10:**
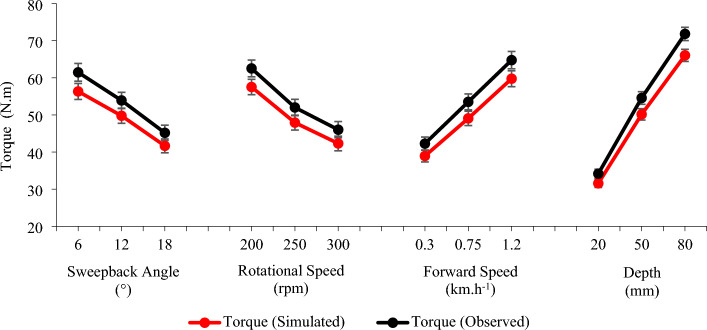
Comparison between the observed and simulated torques for different levels of independent parameters during the single flange rotary tiller experiment.

During the single-flange rotary tiller experiment, the observed and simulated torque values for different levels of independent parameters were compared (Fig. 10). The trend observed during the soil bin experiment for different combinations of independent parameters was reasonably predicted by the DEM simulation experiment. However, in every case, the DEM model underestimated the torque compared to the torque observed during the soil bin experiment.

**Figure 11:**
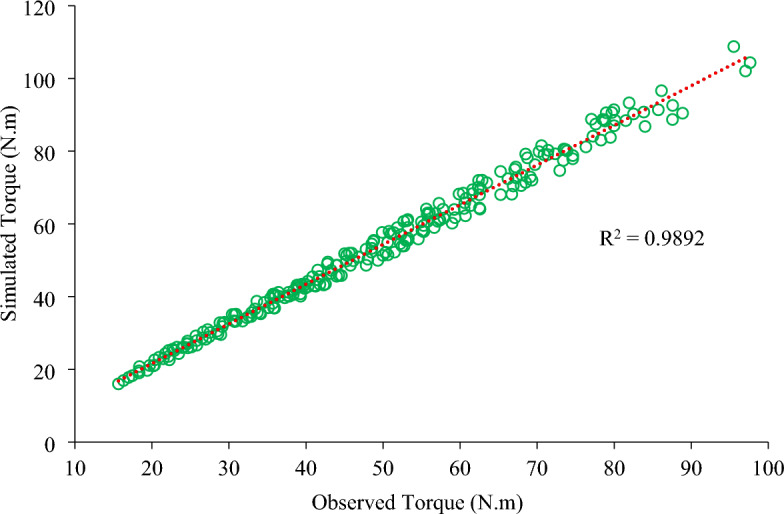
Relationship between the observed and simulated torques during the single-flange experiment.

Figure 11 depicts the relationship between the observed and simulated torque values. There was an excellent correlation (R^2^ ≈ 0.99) between them.

Table 4 shows the relative errors for different levels of independent parameters. The relative error during the experiment varied between 1.29 and 13.51%, with an average value of 7.73%. The results of the validation experiment confirmed its validity for further application.

**Table 4:** Relative error in the simulated torque at different levels of independent parameters.

Sr. no	Independent parameters	Levels	Simulated torque (N.m)	Observed torque (N.m)	Relative error (%)
1	Rotational speed (rpm)	200	57.53 ± 18.87	62.53 ± 20.32	7.89 ± 3.66
		250	47.93 ± 18.05	52.01 ± 19.79	7.61 ± 3.47
		300	42.31 ± 17.75	46.02 ± 19.63	7.69 ± 3.32
2	Sweepback angle (°)	6	56.33 ± 19.38	61.47 ± 21.47	8.15 ± 3.42
		12	49.77 ± 18.28	53.91 ± 19.81	7.55 ± 3.42
		18	41.66 ± 16.93	45.18 ± 18.53	7.48 ± 3.58
3	Forward speed (km.h^-1^)	0.30	38.94 ± 14.47	42.27 ± 15.98	7.57 ± 3.31
		0.75	49.07 ± 17.44	53.53 ± 19.29	8.11 ± 3.33
		1.20	59.76 ± 19.30	64.77 ± 21.17	7.51 ± 3.48
4	Depth (mm)	20	31.56 ± 9.98	34.16 ± 10.92	7.38 ± 3.34
		50	50.15 ± 13.74	54.57 ± 15.33	7.87 ± 3.53
		80	66.05 ± 14.62	71.84 ± 16.03	7.9 3 ± 3.57

## Discussions

The performance of the developed blades was evaluated using a DEM simulation of a single-flange rotary tiller model. This study analyzed several dependent variables, including the average power requirement, average particle velocity (disturbance intensity), and mixing index, to assess the overall performance of the developed blades.

The study revealed that the required torque decreased with increasing rotational speed. However, the number of cuts per unit of time increased as the rotational speed of the rotary tiller increased, leading to an increase in the power requirement. This observation was in agreement with the results obtained by Yang et al.^4^. The power requirement of the blades increased with increasing sweepback angle. This phenomenon might explained by the fact that blades with a sweepback angle of 18° required less soil penetration and throwing torque than blades with sweepback angles of 6° and 12° due to lesser attack surface area. As a result of the lower torque requirement, the 18° sweepback angle blade exhibited a lower power requirement than the other blades. The attack surface area tends to decrease with increasing sweepback angle. Zhao et. al.^11^ and He et al.^20^ reported that the penetration resistance tends to decrease as the attack surface area of the penetrating tool decreases. This might be the reason behind the decreased power requirement with an increase in the sweepback angle. The power requirement was observed to increase with increasing forward speed. This phenomenon can be attributed to the fact that the blade has to handle a greater volume of material as the bite length increases with an increase in forward speed, resulting in a greater power requirement to cut and throw the soil. These findings are in line with the results obtained by Kim et al.^38^. The power requirement is positively related to the depth of operation. It tends to increase rapidly with increasing depth of operation. This rapid increase in average power requirement with an increase in the depth of operation can be attributed to the fact that as the amount of soil to be handled increases with depth, it leads to an increase in power requirement. Torotwa et al.^39^ and Yang et al.^4^ also reported that an increase in the operating depth leads to an increase in the torque requirement, which in turn results in an increase in the power requirement.

The increase in particle velocity with increasing rotational speed can be attributed to the contact force between the particles and the rotary tiller blade increasing with rotational speed. As a result, the momentum generated at the point of impact transfers over a greater distance due to the contacting particles. This displacement causes a greater number of particles to attain a certain velocity and be displaced from their original position. Consequently, the average particle velocity increases with increasing rotational speed of the blade. Similar observations were also reported by Du et al.^26^ while studying the effect of the operating parameters on soil disturbance intensity.

As the sweepback angle increases, the average particle velocity tends to decrease accordingly. The decreased disturbance intensity with increasing sweepback angle can be attributed to the reduced surface area in contact with the blade. As a result, the momentum created in the particles that are in contact with the blade is not transferred over a greater distance, which causes particles farther away from the blade to remain relatively static. This leads to a lower average particle velocity for smaller sweepback angles.

The average particle velocity tends to increase with increasing forward speed. As previously mentioned, the volume of soil particles that are handled by each blade per unit revolution increases as the forward speed of the rotary blade increases. This leads to the transfer of particle momentum over a greater distance, increasing the average particle velocity. Du et al.^26^ reported contradictory results in which they found that disturbance intensity tends to decrease with an increase in forward velocity. However, it is important to note that they focused on spiral horizontal blades and J-shaped rotary blades, while the current study focused on L-shaped blades. This difference in blade types could be a potential reason for the observed contradictory results.

The impact of depth on the volume of particles handled is similar to that of forward speed. The volume of soil particles handled by each blade also tends to increase with increasing depth of operation. As discussed previously, this results in the momentum generated by the blade transferring over a greater distance due to the increased number of neighboring particles in direct contact with the blades. Therefore, an increased depth of operation causes the average velocity of the particles to increase. These results were consistent with results reported by Du et. al.^26^.

The observed increase in the mixing index with increasing rotational speed can be attributed to the fact that a higher rotational speed imparts greater kinetic energy to the soil particles. This increased kinetic energy moves the particles in the top zone into deeper zones. In comparison, particles from deeper zones move into top particle zones, leading to more uniform mixing of the soil particles. The reported findings of Pateriya and Kumar^40^ and Bhambota et al.^41^ are consistent with the observation of an increase in the mixing index with increasing rotational speed. Pateriya and Kumar^40^ reported that the burial performance of rotary tillers increased with increasing rotational speed, which could be attributed to the increased kinetic energy imparted to the soil particles. Similarly, Bhambota et al.^41^ observed an increase in the mixing index with increasing rotational speed for all three blade types (L, J, C) studied. These findings provide further support for the relationship between rotational speed and soil mixing observed in this study.

Due to the reduced effective surface area, the number of particles in contact with the blade and the number of particles thrown by the rotary tiller blade decreased as the sweepback angle increased. Therefore, the number of particles that receive kinetic energy from rotary tiller blades may decrease with increasing sweepback angle. This could be the reason behind the reduction in the mixing index with an increase in the sweepback angle.

The bite length of the rotary tiller blade increases with forward speed. This results in an increase in the volume of soil handled per cut. Consequently, the average kinetic energy transferred to the particles may decrease, leading to restricted movement of the particles to nearby positions. Another reason for the reduced mixing index could be the increased forward speed, which reduces the contact time with the particles. As a result, the momentum in the rotary tiller blades is not efficiently transferred to the particles, and their positions do not change appreciably. The outcomes of this study are in line with the research conducted by Pateriya and Kumar^40^. They found that the mixing burial performance of rotary tillers decreases with increasing forward speed under different field conditions. Hart et al.^42^ also reported similar results while studying the soil amendment incorporation performance of “L”-shaped rotary tiller blades.

The findings concerning the relationship between the mixing index and rotary tiller depth were in line with the observations reported by Salokhe et al.^43^. They reported that deep rotary tilling can be a beneficial option for better mixing of soil. Du et al.^26^ also reported that increased tillage depth improved the burial performance of rotary tiller blades.

The relative error during the experiment varied between 1.29 and 13.51%, while the average relative error observed was 7.73%. Researchers Murray et al.^44^, Sun et al.^45^, Zeng et al.^46^, and Zhao et al.^47^ have reported a similar range of relative errors when studying DEM simulations of soil-tool interactions. Therefore, it can be assumed that the DEM simulation model used in this study yielded reasonably reliable results.

## Conclusions

In this study, the effect of variation of geometric and operational parameters of the modified ‘L’ shaped blade was observed on the power requirement, disturbance intensity, and mixing index. The discrete element method was used to simulate the single flange rotary tiller model with modified blades, and further, it was verified by a soil bin experiment. The following conclusions were drawn from the study.

1. Power requirement was highest for a sweepback angle of 6°, whereas it was lowest for a sweepback angle of 18° across the range of tested operating conditions. Also, the mixing index and disturbance intensity decreased with increasing sweepback angle. A significant decrease in power consumption can be attained with minimal compromise to other performance indicators.
2. A novel method for determining the mixing index in terms of overall mixing index (OMI) and subdomain mixing index (SMI), has the potential to quantify the mixing capability of tillage tools using DEM simulations.
3. The relative error ranged from 1.29% to 13.51%, with an average relative error of 7.73%. This suggests the established DEM simulation model was effective and fairly accurate.

## Data Availability

The datasets used and/or analysed during the current study available from the corresponding author on reasonable request.

## List of symbols

P: Power required by the single flange rotary tiller model (W)
${N}_{r}$: Blade rotational speed (rpm)
${T}_{r}$: Torque acting on the rotary tiller shaft (N.m.)
Q: Number of types of particles
k: Type of particle
${P}_{ki}$: Fraction of particle type k.
${N}_{i}$: Number of particles in a subdomain
M: Number of subdomains
${\text{RE}}_{torque}$: Relative error between the observed and simulated torques (%)
${T}_{\text{o}}$: Observed torque value (N.m.)
${T}_{\text{s}}$: Simulated torque value (N.m.)
